# Validity and Reliability of the Chinese Version of the Care Transition Measure

**DOI:** 10.1371/journal.pone.0127403

**Published:** 2015-05-22

**Authors:** Xiaoyi Cao, Lin Chen, Yongshu Diao, Lang Tian, Wenjie Liu, Xiaolian Jiang

**Affiliations:** 1 Hemodialysis Center, Department of Nephrology, West China Hospital, Sichuan University, Chengdu, Sichuan Province, People’s Republic of China; 2 Department of Nephrology, West China Hospital, Sichuan University, Chengdu, Sichuan Province, People’s Republic of China; 3 Department of hepatobiliary surgery, Sichuan Cancer Hospital, Chengdu, Sichuan Province, People’s Republic of China; 4 Department of Nursing, West China Hospital, Sichuan University, Chengdu, Sichuan Province, People’s Republic of China; Philipps University Marburg, GERMANY

## Abstract

**Background:**

The 15-item care transition measure (CTM-15) is a reliable and valid instrument assessing the quality of care transition from patients’ perspectives. The aim of this study was to evaluate the psychometric properties of the CTM-15 and the CTM-3 (a 3-item short version of the CTM-15) in Mainland China.

**Methodology/Findings:**

This was a cross-sectional study with a convenience sample of 646 patients in a general tertiary-level hospital in Chengdu, China. The results indicated that the Cronbach’s α values of the Chinese version of the two measures were 0.90 and 0.56, and the test-retest reliability values were 0.91 and 0.87, respectively. Three factors were extracted for the CTM-15 in Chinese populations. The CTM-15 and the CTM-3 scores discriminated well between patients with and without re-hospitalization for their index condition. The CTM-15 and the CTM-3 had significant positive relationships with self-rated health status. The CTM-3 score was significantly related to the CTM-15 score, and the CTM-3 score accounted for 64.23% of the variance of the CTM-15 score.

**Conclusions/Significance:**

This study has demonstrated the psychometric properties of the CTM-15 and the CTM-3 in Mainland China. Although the Cronbach’s α value of the CTM-3 is suboptimal, it has exhibited high test-retest reliability, convergent validity and criterion validity. Therefore, the CTM-3 can substitute the CTM-15 as a performance measurement tool when the sample size is large enough to compensate its suboptimal reliability or the reduced response burden is a concern.

## Introduction

Care transition is defined as a series of activities to ensure the coordination and continuity of care for patients who transfer between different healthcare locations or levels of care [1]. Transition from hospital to home is a vulnerable phase in which several challenges exist such as lack of discharge planning and preparation, poor disease prevention and health management abilities, increased post-discharge adverse events, and marked increase in unplanned re-hospitalization and emergency department (ED) visits [2–5]. Care transition is a concern of many healthcare systems because it impacts the overall quality of patients’ care. The Centers for Medicare & Medicaid Services (CMS) proposes that effective and sustainable patient-centered strategies should be developed to improve the quality of care transition [6]. National Quality Forum (NQF) states that accurate and timely transfer of discharge summaries from hospitalists to primary care providers is one of safe practices for high-quality transitional care [7]. Meanwhile, it has been reported that, persistent and efficient communication among patients, caregivers and healthcare professionals is essential for care transition from hospital to home [8].

The 2006 Institute of Medicine (IOM) report “Performance measurement: accelerating improvement” has identified patient-centered transitional care from hospital to home as 1 of 3 priority areas for performance measurement, and has emphasized the necessity and importance to develop a measure evaluating the quality of care transition from patients’ perspective [9]. Patients’ experience is an important element in the measurement of care transition quality as patients and healthcare professionals perform as links among multiple healthcare organizations. However, the report from IOM acknowledges that there is a paucity of instruments assessing the quality of transitional care [8]. The 15-item care transition measure (CTM-15) that focuses on patient-centeredness and care coordination between different healthcare locations has been designed to fill this gap [10]. It has been developed to evaluate the overall care transition experience and not merely the hospital discharge phase. The measure has been demonstrated to be significantly related to a subsequent ED visits and re-hospitalization for the index condition [11]. Moreover, in order to reduce the response burden and facilitate the adoption of the measure for use in public reporting, a 3-item measure of the original 15-item CTM has been developed and tested. Both measures (CTM-15 and CTM-3) have been validated in a diverse population [10], and the later has been endorsed by the NQF and included in the Consumer Assessment of Healthcare Providers and Systems (CAHPS) Hospital Survey in the US in 2010 [12].

Recently, concerns to the quality of care transition have increased in Mainland China due to the severe shortage of healthcare providers and the insufficient health promotion and disease prevention education in our nation’s hospitals and community healthcare centers. Several studies indicated that Chinese patients faced similar problems such as inadequate discharge preparation, lack of self-management knowledge and self-care efficacy, poor medication adherence and increased avoidable re-hospitalization and ED visits during their transitions from hospital to home [13–15]. However, no studies have used validated measures to evaluate the care transition process. Thus, it is essential to develop an instrument to measure patients' experience of their transitions from hospital to home in Mainland China.

The CTM-15 and CTM-3 have been demonstrated as valid tools in many English-speaking and Spanish-speaking countries. However, whether these two measures can be used among patients in different social, ethnic and cultural backgrounds need to be further tested. Therefore, the aim of the study was to evaluate the psychometric properties of the CTM-15 and CTM-3 among patients in Mainland China.

## Methods

### Samples

This was a cross-sectional study with a convenience sampling method used to select patients in a general tertiary-level hospital in Chengdu, China. The inclusion criteria were as follows: (1) aged 18 and older; (2) could be contacted by mobile phones or emails after discharge; (3) received hospital care from disciplines of general medicine, geriatric medicine or oncological medicine, and returned to home residence not long-term care facilities after discharge; (4) agreed to participate in the study. Patients who had visual or hearing impairment, mental disorder or dementia were excluded. The final sample included 646 patients with a response rate of 92.3%.

### Measures

#### Care Transition Measure-15 (CTM-15)

This scale assesses the extent to which patients are being prepared to participate in self-management behaviors after discharge and evaluates the overall quality of care transition from patients’ perspective. The measure includes 15 items and 4 dimensions (critical understanding, importance of preferences, management preparation and existence of a written and understandable care plan). It is constructed as a second-order factor structure in which 15 items each belong to 1 of the 4 subscale and the 4 subscales make up the over arching unidimensional construct evaluating the overall quality of transitional care and summarized as one total score. The instrument is evaluated by a four-point scale ranging from 1 *“strongly disagree”* to 4 *“strongly agree”*, and the initial total score will be linearly transformed to a score on a 0–100 scale with higher scores indicating better care transition quality [10]. The Cronbach’s α value of the measure is 0.93. It has good discriminant validity with statistically significant differences in the CTM-15 scores found between patients who re-hospitalized or visited to ED and those who did not. The results of confirmatory factor analysis (CFA) demonstrates an excellent construct validity of the second-order factor structure of the 4-factor measure (χ2 = 169.73; *p* = 0.46; Comparative Fit Index [CFI] = 0.95; Tucker Lewis Index [TLI] = 0.99; Weighted Root-Mean-Square Residual [WRMR] = 1.15) [11].

#### Care Transition Measure-3 (CTM-3)

The measure is a short version of the CTM-15. It is composed of 3 items which are derived from critical understanding and importance of preferences subscales for the CTM-15: (1) having patients and their family or caregivers’ preferences incorporated into health care plan (item 2); (2) understanding post-discharge health self-management activities (item 9); and (3) understanding the purpose for taking medications (item 13). The scale has reduced response burden. The measure is shown to discriminate between patients discharged from the hospital who did and did not have a subsequent ED visit or re-hospitalization for their index condition. It is significantly related to the CTM-15 and accounts for 88.0% of the variance of the CTM-15 score, indicating a high criterion validity [11]. The scale also shows a high convergent validity with significant associations with health status and care experiences after discharge, respectively [16,17].

In addition, demographic characteristics such as gender, age, unplanned ED visits and re-hospitalization for the index condition were also collected.

### Translation procedure

A forward-back-translation procedure was performed in our study. First, a graduate in nursing science translated the English version into Chinese, and a registered nurse with a doctoral degree in nursing science who graduated from a university in Hong Kong modified the translated version. Second, a graduate majoring in English linguistics in Australia and a registered nurse in the US translated the Chinese version into English. Finally, a professor in nursing science who graduated from a US university compared the backward translation with the original version, and confirmed the conceptual and literal equivalence of the Chinese version. Moreover, thirty-two patients with cardiovascular diseases were recruited for the monolingual test. The results supported the readability, comprehensibility and cultural adaptation of the Chinese version. The findings also showed that all the participants understood the items easily and took 20 minutes to complete the questionnaire.

### Data collection

Nine experts including two professors in cardiology, two associate professors in respirology, an associate professor in oncology, three clinical nursing specialists in the disciplines of general medicine, geriatric medicine or oncological medicine, and a professor in chronic disease management were invited to evaluate the content validity of the Chinese version. Item content validity was evaluated by the item content validity index (I-CVI). Clarity of phrasing and applicability of content were used as criteria for the item content validity assessment [18], which is evaluated by a four-point scale ranging from 1 *“not relevant”* to 4 *“highly relevant”* [19].

Prior to the study, three graduates each with a master’s degree in medical science were recruited and trained as research assistants. First, patients were told the purpose and importance of the study and a written informed consent was obtained from each participant before the study. Then, patients were contacted by emails or mobile phones to complete the questionnaires4 weeks after their discharge. With regard to the patients who could be contacted by emails, they were required to complete the web-based questionnaires according to their actual feelings and return the answers in a week. As for the patients who could be contacted by mobile phones, the research assistants read the questions and responses word for word and recorded their answers. In addition, 35 patients were randomly selected from the 646 patients to complete the questionnaires 2 weeks later, and the test-retest reliability was examined. This survey was conducted from March 1, 2014 to May 30, 2014.

### Data analyses

The statistical analysis packages used in the study were SPSS 16.0 and Amos 18.0 (SPSS Inc., Chicago, IL, USA). As our data was not normally distributed, item-total correlation was calculated by Spearman correlation analysis. Items with higher item-total correlation values (r > 0.30) and statistical significance (*p* < 0.001) were considered as having desirable discriminating power [20,21]. Cronbach’ α was used to assess the internal consistency reliability. Spearman correlation with a 2-week interval between evaluations was conducted to calculate the test-retest reliability. The I-CVI was calculated as the number of experts giving a rating of either “quite relevant” or “highly relevant”, divided by the number of experts. The scale CVI (S-CVI) can be calculated as the average of the I-CVI for all items on the scale that achieved ratings of “quite relevant” or “highly relevant” [22]. As the data in the study did not follow a normal distribution, the bootstrap method was performed to test the stability of the psychometric properties of the Chinese version of the CTM-15. Model fit was assessed using a combination of fit indices including χ2/df, Goodness of Fit Index [GFI], Adjusted Goodness of Fit Index [AGFI], Comparative Fit Index [CFI], Tacker-Lewis Index [TLI], Root Mean Square Error of Approximation [RMSEA] and Standardized Root Mean Square Residual [SRMR]. The values of χ2/df ranging from 1 to 2, GFI > 0.90, AGFI > 0.90, CFI > 0.90, TLI ≥ 0.95,RMSEA ≤ 0.06 and SRMR ≤ 0.08 were regarded as acceptable model fit [23,24]. Exploratory factor analysis (EFA) was conducted to assess the factor structure of the translated CTM-15. For the evaluation of construct validity, Mann-Whitney U tests were performed to evaluate whether patients who visited ED or re-hospitalized for their index condition after discharge would have lower CTM scores than those who did not (known-groups validity). Spearman correlation coefficients between the two translated measures and the health status measured by the SF-12v2 subscales of self-reported physical and mental health [25] were used to assess the convergent validity. For the testing of the criterion validity of the CTM-3, correlation analysis between the CTM-3 and the full CTM-15 was conducted. *P* < 0.05 was considered statistically significant.

### Ethical statement

Written informed consent was obtained from each participant who was assured of confidentiality, anonymity and right to withdraw from this study at any time. Ethical approval was obtained from the Human Subjects Ethics Sub-committee of Sichuan University prior to the survey.

## Results

Of the 646 respondents in the study, 76.8% were men and 23.2% were women. The age ranged from 39 to 90 with the average age of 69.5 years (SD = 8.1). Most of the respondents were married (88.1%). 59.7% of the respondents graduated from senior high school or above. Almost all the respondents had health insurance (98.5%). As for the types of diseases, 28.5% of the respondents suffered from cardiovascular disease and 14.9% experienced diabetes mellitus. The Charlson comorbidity index score ranged from 0 to 6 with the Huber´s M estimator of 1.4. The rates of re-hospitalization or ED visits for the index condition after discharge were 7.0% and 3.5%, respectively (Table 1).

**Table 1:** Characteristics of samples (n = 646).

Variables		
Gender, n (%)	Male	496 (76.8)
	Female	150 (23.2)
Age (years)	Mean (SD)	69.5 (8.1)
	Range	39–90
Marital status, n (%)	Married	570 (88.1)
	Single/divorced/widowed	76 (11.9)
Educational level, n (%)	Primary school or under	134 (20.6)
	Junior high school	144 (22.2)
	Senior high school	140 (21.6)
	College or university and above	228 (35.6)
Types of diseases, n (%)	Cardiovascular disease	184 (28.5)
	Diabetes mellitus	96 (14.9)
	Chronic kidney disease	84 (13.0)
	Respiratory disease	66 (10.2)
	Cancer	114 (17.6)
	Other	122 (18.8)
Health insurance, n (%)	No	10 (1.5)
	Yes	636 (98.5)
	Range	2–22
Re-hospitalization for the index condition, n (%)	No	600 (93.0)
	Yes	46 (7.0)
Visits to emergency department for the index condition, n (%)	No	624 (96.5)
	Yes	22 (3.5)

The results indicated that the item-total correlation values ranged from 0.46 to 0.70, which suggested desirable discriminating power of the items in the CTM-15. The Cronbach’s α of theCTM-15 and the CTM-3 were 0.90 and 0.56, and the test-retest reliability values were 0.91 and 0.87, respectively, which demonstrated good stability of these two measures over time. With regard to the validity analysis, the I-CVI of the CTM-15 ranged from 0.89 to 1.00 and the S-CVI was 0.99.Both the I-CVI and the S-CVI of the CTM-3 were 1.00, indicating adequate content validity.

Moreover, CFA was conducted to test the 4-factor CTM-15. As presented in Table 2, the results indicated that, the standardized factor loading values of the 15 items ranged from 0.47 to 0.89 (*p* < 0.05) and the squared correlations ranged from 0.22 to 0.79. The AVE (average variance extracted) of each factor ranged from 0.34 to 0.58. Meanwhile, the findings showed that the 4-factor model did not have a good fit to the data in our study (χ2/df = 7.49, *p* < 0.001, GFI = 0.89, AGFI = 0.85, CFI = 0.88, TLI = 0.85, RMSEA = 0.10 and SRMR = 0.08) (Table 2). Next, the principal components analysis with promax rotation method was performed to extract the factor structure of the CTM-15. The Kaiser-Meyer-Olkin (KMO) measure of sampling adequacy was 0.88, and Bartlett’s test of sphericity was statistically significant (χ2 = 4619.80, df = 105, *p* < 0.001), which supported the use of EFA as an appropriate procedure. Three factors were extracted with eigenvalues >1.00, which explained 59.26% of the total variance. Factors with loading weights > 0.40 were presented in Table 3. These extracted factors were named as health management preparation (factor 1), medication management preparation (factor 2) and importance of preferences (factor 3). The findings indicated that the item 7 (having a written care plan) and the item 12 (having a written list of appointments and tests) which belonged to the existence of a written and understandable care plan subscale originally had greater loadings on the health management preparation subscale. The items 13–15 (understand medications’ purpose, usage and side effects) in the critical thinking subscale of the original measure belonged to the medication management preparation subscale in the translated version (Table 3).

**Table 2:** Results of confirmatory factor analysis of the CTM-15 (n = 646).

	Factor loadings	Squared correlations	Standard error of variances
Factor 1: Critical understanding, AVE = 0.40			
Item 9. Good understanding of things I was responsible for	0.60	0.36	0.64
Item 10. Confident I knew what to do	0.77	0.59	0.41
Item 11. Confident could do what needed	0.64	0.41	0.59
Item 13. Understand medications’ purpose	0.60	0.36	0.64
Item 14. Understand how to take medications	0.59	0.35	0.65
Item 15. Understand medications’ side effects	0.55	0.30	0.70
Factor 2: Importance of preferences, AVE = 0.41			
Item 1. Agreed health goals and means	0.82	0.67	0.33
Item 2. Preferences deciding health care needs	0.47	0.22	0.78
Item 3. Preferences deciding where needs met	0.58	0.34	0.66
Factor 3: Management preparation, AVE = 0.58			
Item 4. Had information needed for self-care	0.88	0.77	0.23
Item 5. Understand how to manage health	0.89	0.79	0.21
Item 6. Understand signs and symptoms	0.65	0.42	0.58
Item 8. Understand what makes better or worse	0.57	0.32	0.68
Factor 4: Existence of a written and understandable care plan, AVE = 0.34			
Item 7. Had written care plan	0.65	0.42	0.58
Item 12. Had written list of appointments and tests	0.51	0.26	0.74
Model fit			
χ2 (*df* = 82), *p* < 0.001	614.44		
χ2/df	7.49		
GFI	0.89		
AGFI	0.85		
CFI	0.88		
TLI	0.85		
RMSEA	0.10		
SRMR	0.08		

AVE: average variance extracted, GFI: goodness of fit index, AGFI: adjusted goodness of fit index, CFI: comparative fit index, TLI: Tacker-Lewis index, RMSEA: root mean square error of approximation, SRMR: standardized root mean square residual.

**Table 3:** Results of exploratory factor analysis of the CTM-15 (n = 646).

	Factor loadings	Factor loadings	Factor loadings
Factor 1: Health management preparation			
Item 4. Had information needed for self-care	0.70		
Item 5. Understand how to manage health	0.76		
Item 6. Understand signs and symptoms.	0.63		
Item 7. Had written care plan	0.67		
Item 8. Understand what makes better or worse	0.72		
Item 9. Good understanding of things I was responsible for	0.50		
Item 10. Confident I knew what to do	0.60		
Item 11. Confident could do what needed	0.49		
Item 12. Had written list of appointments and tests	0.50		
Explained variance: 42.62%			
Factor 2: Medication management preparation			
Item 13. Understand medications’ purpose		0.83	
Item 14. Understand how to take medications		0.77	
Item 15. Understand medications’ side effects		0.65	
Explained variance: 7.94%			
Factor 3: Importance of preferences			
Item 1. Agreed health goals and means			0.72
Item 2. Preferences deciding health care needs			0.86
Item 3. Preferences deciding where needs met			0.82
Explained variance: 7.24%			
Cumulative explained variance: 59.26%			

With regard to the known-group validity analysis, the findings showed that statistically significant differences in the CTM-15 and the CTM-3 scores were found between patients who re-hospitalized for their index condition and those who did not. However, patients who reported ED visits for the index condition did not have significant lower CTM-15 and CTM-3 scores compared to those who did not (Table 4).

**Table 4:** Scores on the CTM-15 and CTM-3 among different subgroups (n = 646).

Variables	n	CTM-15	CTM-3
		Mean (SD)	Mean (SD)
Re-hospitalization for the index condition			
No	600	76.69(17.25)	71.57(21.06)
Yes	46	62.91(21.27)	60.29(22.55)
*P* value (Z statistics/Effect size)		0.002 (4.818/0.174)	0.010(4.153/0.163)
Visits to emergency department for the index condition			
No	624	75.55 (17.48)	70.77 (21.24)
Yes	22	61.81 (29.82)	63.97(18.88)
*P* value (Z statistics/Effect size)		0.051(2.863/0.113)	0.064(2.619/0.103)

In addition, for the testing of convergent validity, the findings revealed that both the CTM-15 and the CTM-3 had significant positive relationships with the self-rated physical health (r = 0.31, *p* < 0.001; r = 0.28, *p* < 0.001) and mental health (r = 0.27, *p* < 0.001; r = 0.33, *p* < 0.001). The results demonstrated acceptable convergent validity of the two measures.

For the criterion validity of the CTM-3, the results revealed that the CTM-3 score had a significant positive relationship with the CTM-15 score (r = 0.78, *p* < 0.001). The CTM-3 score accounted for 64.23% of the variance of the CTM-15 score, demonstrating good criterion validity of the CTM-3.

## Discussion

With regard to the reliability analysis, we found that the Cronbach’s α value of the CTM-15 was 0.90, which is in parallel to the results of previous studies [11,16,17,26]. It was reported that a measure would be reliable if its Cronbach’s α value exceeded 0.80 [27]. Our results demonstrated a favorable internal consistency reliability of the CTM-15. However, the Cronbach’s α value of the CTM-3 was 0.56 in the present study, which is similar to the finding of a previous study in Singapore with a Cronbach’s α value of 0.58 [17]. It was known that the Cronbach’s α value reduced with the decreased number of items. Therefore, it was not surprised that the Cronbach’s α value for the CTM-3 was lower than those for the full CTM-15.

We also found that the test-retest reliability of the CTM-15 and the CTM-3 were 0.91 and 0.87, respectively, which indicated the stability of these two measures over time. The test-retest reliability over 0.80 indicated good reliability [28]. However, the 2-week interval used to calculate the test-retest reliability in our study may result in over-estimation due to the respondents’ memory of their first responses. Thus, careful explanations should be used for the results. Further studies for the test-retest reliability analysis should include a longer time interval and larger sample size.

As for the content validity assessment, endorsement was obtained with a minimum I-CVI of 1 for a panel of 5 members or less and 0.80 for a panel of 6 members or more [29]. Meanwhile, the S-CVI ≥ 0.90 was considered good [21,30]. In our study, nine experts were invited, and the I-CVI and the S-CVI of the two measures exceeded 0.80, which suggested good content validity.

Moreover, the findings of CFA revealed that the second-order factor structure of the original English version of the CTM-15 did not have a good fit to the data in our study. And the results of EFA indicated that three factors were extracted from the translated version, which is not consistent with the original version comprising 4 factors [10]. The item 7 (having a written care plan) and the item 12 (having a written list of appointments and tests) which belonged to the existence of a written and understandable care plan subscale originally were incorporated into the health management preparation factor in the present study. The difference may be attributed to the dissimilar healthcare policy in Mainland China and in the US. It is reported that, in the US, hospital discharge summaries are usually transmitted from hospitalists to primary care physicians directly or handed to the patients who perform as couriers. It is the responsibilities of primary care providers for discharge information delivery and management. However, in Mainland China, hospital discharge summaries are directly transmitted to the patients who are responsible for their post-discharge disease prevention and health management themselves. Primary care physicians cannot obtain hospital delivery information for patients until their visits to community healthcare centers. Furthermore, we found that the items 13–15 (understand medications’ purpose, usage and side effects) in the critical thinking subscale of the original measure belonged to a separate factor (medication management preparation) in the translated version. That may be ascribed to the fact that the Chinese patients pay more attention to medication management than to other healthcare behaviors such as diets and exercises after discharge. The results of a qualitative interview in our study suggested that adherence to medication was a top priority compared to other healthcare compliance matters. In the present study, we also found that the items 9–10 (understanding of things I was responsible for and being confident I knew what to do), the items 4–6 and the item 8 (having information needed for self-care, understanding how to manage health, understanding signs and symptoms and understanding what makes better or worse), as well as the item 7 (having a written care plan) and the item 12 (having a written list of appointments and tests) which belonged to critical thinking, management preparation and existence of a written and understandable care plan subscales originally were combined as one factor (health management preparation)in the translated version. That may be related to their similarities in emphasizing the understanding and confidence in health management among patients after discharge. In addition, it is essential and important to note that the CTM-15 is scored as a unidimensional structure, therefore, the domain-level structure does not have direct impact on the scoring [11].

With regard to the known-group validity evaluation, our findings demonstrated that patients who re-hospitalized for their index condition had significant lower CTM-15 and CTM-3 scores than those who did not, which is similar to the results of earlier studies [16,17,31]. It is suggested that the factors that relate to re-hospitalization in patients included three types: (1) the patient's characteristics such as language or culture barrier, and poor medication adherence; (2) the clinician's characteristics such as inappropriate discharge planning or medication; (3) the characteristics of the hospital care system such as lack of post-discharge follow-up, inadequate patient education and lapse in discharge summary delivery from hospitalists to primary care providers [32]. Furthermore, it was reported that, the reasons for re-hospitalization included inadequate discharge teaching, poor hospital discharge process and information transmission procedure [33]. Therefore, patients who re-hospitalized may receive inadequate discharge information and self-care knowledge and skills when they transferred from hospital to home, in last, result in poor care transition quality. However, no statistically significant differences in the CTM-15 and the CTM-3 scores were found between patients who visited ED and those who did not. It may be partly ascribed to the sampling error supported by the observation that less than thirty patients reported ED visits in Chinese patients. It is possible that those patients visited ED for reasons not related to poor quality of care transition.

With regard to the convergent validity analysis, our findings indicated that both the CTM-15 and the CTM-3 had significant positive associations with self-rated physical and mental health, which is in parallel to the results of previous studies. Shadmi et al. (2009) demonstrated the significant and positive correlations between the total CTM-15 score and self-reported physical and mental health in the Hebrew sample [16]. Similarly, Blendon (2003) reported that adults with poorer health status had more unmet needs for care transition [34].

In addition, we found that the CTM-3 had a significant positive association with the CTM-15, which is similar to the results of previous studies. It was reported that the CTM-3 score were significantly related to the CTM-15 score for patients in different cultural and social backgrounds [16,17]. Nevertheless, the correlation value between the two measures in our study was relatively lower (0.78) than those for patients in Singapore (0.89) and those for cancer patients in Israel (0.87) [16,17]. The CTM-3 has been advised to public reporting with a larger sample size. Possible explanations for the observed data in our study may be attributed to the smaller sample size or the different samples.

Several limitations are identified in the study. First, an acquiescence bias existed in the study (5.7% respondents replied "Strongly agree" to all items). Second, a convenience sampling method was used and patients were recruited from a general tertiary-level hospital in one area in China, which might undermine the sample representativeness and limit the generalizability of our findings. Future studies including more patients in other areas in Mainland China are needed. Finally, as for the validity tests, the content validity, construct validity and convergent validity have been evaluated in the study. Future studies should evaluate the measures’ predictive validity.

Despite these limitations, the CTM-15 has been demonstrated as a reliable and valid measure evaluating care transition quality among patients in Mainland China. Although the Cronbach’s α value of the CTM-3 is suboptimal, it has exhibited favorable test-retest reliability, convergent validity and criterion validity. Thus, the CTM-3 can replace the CTM-15 as an effective performance measurement tool when the sample size is large enough to compensate its suboptimal reliability or the reduced response burden is a concern.

## Supporting Information

S1 Dataset (XLS)
